# Promoting Local Ownership: Lessons Learned from Process of Transitioning Clinical Mentoring of HIV Care and Treatment in Ethiopia

**DOI:** 10.3389/fpubh.2018.00014

**Published:** 2018-02-05

**Authors:** Getnet M. Kassie, Teklu Belay, Anjali Sharma, Getachew Feleke

**Affiliations:** ^1^Addis Ababa University, Addis Ababa, Ethiopia; ^2^I-TECH Ethiopia, Addis Ababa, Ethiopia; ^3^Liverpool School of Tropical Medicine, Liverpool, United Kingdom

**Keywords:** clinical mentoring, transition, HIV care and treatment, multidisciplinary teams, ownership, sustainability

## Abstract

**Introduction:**

Focus on improving access and quality of HIV care and treatment gained acceptance in Ethiopia through the work of the International Training and Education Center for Health. The initiative deployed mobile field-based teams and capacity building teams to mentor health care providers on clinical services and program delivery in three regions, namely Tigray, Amhara, and Afar. Transitioning of the clinical mentoring program (CMP) began in 2012 through capacity building and transfer of skills and knowledge to local health care providers and management.

**Objective:**

The initiative explored the process of transitioning a CMP on HIV care and treatment to local ownership and documented key lessons learned.

**Methods:**

A mixed qualitative design was used employing focus group discussions, individual in-depth interviews, and review of secondary data. The participants included regional focal persons, mentors, mentees, multidisciplinary team members, and International Training and Education Center for Health (I-TECH) staff. Three facilities were selected in each region. Data were collected by trained research assistants using customized guides for interviews and with data extraction format. The interviews were recorded and fully transcribed. Open Code software was used for coding and categorizing the data.

**Results:**

A total of 16 focus group discussions and 20 individual in-depth interviews were conducted. The critical processes for transitioning a project were: establishment of a mentoring transition task force, development of a roadmap to define steps and directions for implementing the transition, and signing of a memorandum of understanding (MOU) between the respective regional health bureaus and I-TECH Ethiopia to formalize the transition. The elements of implementation included mentorship and capacity building, joint mentoring, supportive supervision, review meetings, and independent mentoring supported by facility-based mechanisms: multidisciplinary team meetings, case-based discussions, and catchment area meetings.

**Conclusion:**

The process of transitioning the CMP to local ownership involved signing an MOU, training of mentors, and building capacity of mentoring in each region. The experience shed light on how to transition donor-supported work to local country ownership, with key lessons related to strengthening the structures of regional health bureaus, and other facilities addressing critical issues and ensuring continuity of the facility-based activities.

## Introduction

Sustaining donor-supported investments over time requires well-designed strategies to strengthen local ownership, assure monitoring and documentation, engage local providers, and commit to structural accommodations and resource allocation (1, 2). Given the devastating effects of HIV/AIDS in many developing countries, several intervention programs were initiated and implemented with limited contextual exploration and corresponding learning tools. In Sub Saharan Africa (SSA), the demand for services often surpasses available resources and is constrained by lack of capacity and inefficient management. Under these circumstances, host governments are mostly confronted with local challenges versus desire to scale up interventions to reach the needy. This is further compounded by prevailing limited evidence for planning and expanding HIV/AIDS interventions (3–5).

It has been demonstrated that scaling up of antiretroviral treatment required corresponding knowledge and skills of provision of HIV care and managing services in SSA where the burden of HIV/AIDS is greatest (6, 7). This called for introduction of non-governmental organizations to SSA with new strategies to build local capacity, generate evidence, and enhance scale up of HIV/AIDS prevention, care, and treatment services. As part of this effort renowned United States universities have been taking part in HIV/AIDS care and treatment services in Ethiopia for over a decade.

From 2003 to 2005, International Training and Education Center for Health (I-TECH) affiliated with University of Washington and University of California, San Francisco provided technical assistance to the Federal Ministry of Health and the Federal HIV/AIDS Prevention and Control Office of Ethiopia. The Technical Assistance encompassed training of health care workers in clinical services and program delivery, and related capacity building activities. In 2005, following the US President’s Emergency Plan for AIDS Relief (PEPFAR) initiative to scale up Ethiopia’s antiretroviral therapy (ART) program, I-TECH Ethiopia focused on strengthening HIV prevention, care, and treatment services in three northern administrative regions namely Afar, Amhara, and Tigray (8–10).

The Federal Ministry of Health of Ethiopia promoted use of clinical mentoring to expand comprehensive HIV prevention, care, and treatment to ensure universal access to care in the country. Thus, the work of I-TECH expanded from that of primarily training health care workers to providing Technical Assistance (with PEPFAR funding) for the rollout of the ART program. The Technical Assistance was provided through deploying mobile field-based teams that mentored health care providers in clinical service and program delivery. The clinical mentoring program (CMP) was conducted in 48 facilities of the three regions. These include 17 hospitals in Amhara, 17 facilities in Afar, and 14 hospitals in Tigray regions. In Afar, the number of hospitals is few (5). Therefore, hospitals and high-load health centers were included in the CMP. Twelve of the 48 facilities were categorized as lead and the remaining 36 as cluster facilities. Lead hospitals are staffed with skilled clinical staff that can mentor and supervise cluster facilities.

In 2012, I-TECH initiated preparatory activities for transitioning the CMP to the regional health bureaus. This required a change from direct mentoring to supporting locally administered mentoring programs. Most of the field-based teams were replaced by capacity building teams. The capacity building team worked with the regional health bureau in developing the locally administered mentoring program. The transition plan was within the PEPFAR’s intentions and strategic direction to promote local ownership and sustain the HIV/AIDS prevention, care, and treatment program (5).

According to the World Health Organization (WHO), clinical mentoring is “a system of practical training and consultation that fosters ongoing professional development to yield sustainable high-quality clinical care outcomes” (11). In clinical mentoring, clinicians with substantial expertise in ART and the management of opportunistic infections mentor less-experienced HIV clinical care and treatment providers. This is done through responding to questions, reviewing clinical cases, providing feedback, and assisting in case management on an ongoing basis (12). However, all these program activities require resources and skills in mentoring and program administration.

The model began with building knowledge and skills through didactic training. This was followed by immediate practice of new skills under close supervision. The training was conducted in the mentee’s own clinical setting, together with provision of one-on-one mentoring by specialists and/or experienced clinicians. I-TECH Ethiopia’s field teams were engaged in clinical mentoring and systems strengthening to address critical knowledge, skill, and systems gaps. Health care providers gain confidence through guided practice, and incrementally assume greater responsibility and decision-making in the provision of ART. Later, the field-based teams were reduced in number and capacity building teams were established and worked toward enhancing the transition of CMP through capacity building. Eventually, health care providers are expected to function independently, seeking guidance, and advice from their mentors only as needed. Ultimately, the health bureau is expected to own and lead the CMP.

There are few published reports documenting best practices of transitioning HIV/AIDS clinical mentorship programs in SSA. Exploring and documenting the process of transitioning ownership of programs such as this would add to the body of knowledge in the field. The lessons could be adapted to similar collaborative programs that are spear headed by foreign non-governmental organizations.

The objective of this assessment is to explore the process of transitioning CMP to promote local ownership in three intervention regions of Ethiopia.

## Materials and Methods

### Study Design

We employed a qualitative research method that included review of secondary data to assess the CMP transitioning in Afar, Amhara, and Tigray regions. Three facilities, one lead (hospitals with first line mentors) and two cluster facilities (mentored by mentors from lead facilities) from each region (for a total of nine) were selected. The selection was made using purposive sampling considering logistics, distribution of facilities, and in consultation with regional health bureaus. From lead facilities, multidisciplinary team members, government mentors, and mentees were chosen; from cluster facilities, multidisciplinary team members, and mentees were selected. In addition, the regional health bureaus mentoring focal person from each region, I-TECH capacity building team members, regional directors, and regional planning, monitoring, and evaluation (RPM&E) coordinators were included. Allocation of participants for focus group and in-depth interview considered mix and availability of sufficient number of discussants for focus groups and relevance of positions in running the CMP program.

### Data Collection and Tools

Three teams consisting of two people collected the data. The arrangement was that one was a moderator and the other a note taker. Focus group discussions and individual in-depth interviews were conducted to collect the data. Furthermore, secondary data were collected from I-TECH’s head office, regional health bureaus, and facilities.

Customized guides were prepared for the different groups of study participants. These include a mentee interview guide; a lead facility mentors’ guide; a regional health bureau mentoring focal person interview guide; and a focus group discussion guide for multidisciplinary teams at cluster and lead facilities, capacity building teams, and I-TECH regional directors and RPM&E coordinators. The purpose of the guide was to determine the progress of the CMP transition at each level, including the degree of preparation at regional health bureaus and facilities for making the transition, and awareness and knowledge of transition-related issues—e.g., transition goals, facility engagement, challenges, I-TECH’s role in the transition, and recommendations.

Questions were translated and administered in Amharic; it was thought that participants would be more at ease during discussions and interviews than they would if discussions were conducted in English. Although the questions were translated and forwarded in Amharic, the English words used in day-to-day practice, such as *clinical mentoring, lead hospital, cluster hospital*, etc. were maintained and used in the interviews.

All interviews and discussions were recorded using digital audio recorders. Recordings were made only after obtaining consent from study participants. Discussion sites were selected in facilities and time arrangements were based on convenience to health workers in the facilities. In all the sites interview and groups’ discussion, rooms were identified by the team along with heads of the respective health facilities.

For the secondary data, we used a data extraction format. Other data sources, such as 2-year I-TECH monitoring and evaluation reports were reviewed.

### Data Collectors Training and Pre-Testing

A total of six evaluating assistants were recruited and trained for 5 days. All of them had previous experience in qualitative data collection methods, and possessed the required levels of education for their respective positions. Training of the evaluation teams included discussions on the tools, field testing, focus group and interview methods, instruction on how to use a digital audio recorder, and simulating exercises. Pre-testing took place at Debre Berhan Hospital located about 130 km northeast of Addis Ababa.

### Data Labeling, Entry, and Analysis

Data were systematically labeled, with labels listing name of region, qualitative method used, name of facility, and date of collection. Each audio recording was listened to, fully transcribed, and then translated into English. Translations were checked and edited between the transcriber and team leader. Additionally, field notes, summary notes, and other relevant documents were gathered. The report team lead listened to the recordings and studied the data, then checked each transcription against its corresponding recording.

All documents—including transcriptions, field notes, summary notes, audio records, reports, and other documents—were entered into the computer. Transcriptions were checked and converted into text format, then data files were created using Open Code qualitative software. The data set included a total of 36 text files. Each file was coded line-by-line, then the codes were categorized. Using a content analysis approach, the categories were synthesized into themes. As described earlier, the themes reflected the predesigned guide and data abstraction. However, not all themes were bound to the guide.

The documents collected from I-TECH, the regional health bureaus, and health care facilities, were reviewed. The review included background information, capacity building activities, evidence of local ownership, managerial and implementation capacity, and related successes and challenges.

### Ethical Considerations

The protocol has obtained clearance from the Federal Ministry of Health, the respective regional health bureaus in Ethiopia and Unites States Center for Disease Control and Prevention (CDC). Ethical principles were respected, and participants were fully informed of the study and asked to give consent through reading an information sheet.

## Results

Data collection took place from August 8 to 25, 2014. We conducted 16 focus group discussions and 20 individual in-depth interviews in, Amhara, Afar, and Tigray regions. A total of 122 people participated in the focus groups and interviews. Details are indicated in Table 1.

**Table 1:** Focus groups (*n* = 16) and interviews (*n* = 20) by region.

Target group (format)	No. of participants by region			Total
	Tigray RHB	Amhara RHB	Afar RHB	
Regional focal person (interview)	1	1	1	3

I-TECH Capacity Building Team (focus group)	1	1	1	3

I-TECH regional directors and RPM&E coordinators (focus group)				1

Mentors (focus group)	1	1	1	3
	Axum Hospital	Debremarkos Hospital	Dubti Hospital	

Multidisciplinary teams (focus group)	3	3	3	9
	Shire-Suhul Hospital	Debretabor Hospital	Dubti Hospital	
	Axum Hospital	Debremarkos Hospital	Awash Health Center	
	Wukro Hospital	Finoteselam Hospital	Worer Health Center	

Mentees (interview)	6	5	6	17
	Shire-Suhul Hospital	Debretabor Hospital	Dubti Hospital	
	Axum Hospital	Debremarkos Hospital	Awash Health Center	
	Wukro Hospital	Finoteselam Hospital	Worer Health Center	

Using content analysis, categories were organized into three themes where issues of the transition are presented in detail. The first theme explored the level of commitment by the regional health bureaus to own the CMP; the second theme elaborated the degree of preparation/readiness by regional health bureaus to own the CMP including structural arrangements and local capacity. The third theme focused on the degree of preparation and/readiness by health facilities to own the CMP.

### Commitment to Own the CMP

Study participants were asked to discuss whether it was a good idea to hand over the CMP. There was a consensus in responding to this question in general terms. Almost all the focus group participants and interviewees believed it was necessary that the program be owned by the regional health bureaus.

It started earlier, in November 2012. You cannot always expect everything from partners. We started to fully implement in 2014. We have trained potential mentors and have been operating independently since July.—Interview with regional focal person

In support of the results from the discussants and interviews, results from review of the secondary data showed that there were two key documents that were used to translate the CMP transition (1) roadmap for implementing the transition which was developed by a taskforce and (2) memorandum of understanding (MOU) signed by the regional health bureaus and I-TECH (8–11). Although I-TECH has been involved in clinical mentoring since 2005, the shift from direct mentoring to local capacity building in mentoring intensified during the last 2 years, i.e., since the annual donor mandated Country Operation Plan 2012 (COP12).

Formerly, the focus was on clinical care for patient treatment; during the transition, the focus has been on the program.—regional health bureau focal person

Under PEPFAR II strategic direction, I-TECH started partial mentoring transition to initiate smooth transitioning of CMP. As part of this process, it reduced its field-based team staff by 50%, and established capacity building teams to enhance local capacity and promote ownership in the respective regions. The following statement from a focus group participant illustrates the relevance of transitioning.

…they have been making good things for us; the transition is good for continuing the program, because the work/program would not have continuity if we were always dependent on others.—Focus group discussion with multidisciplinary team, hospital-C2

### RHB Readiness to OWN CMP

The process in which the regional health bureaus have been taking over the CMP (with assistance from I-TECH) is presented in the following subthemes.

### Structural and Functional Preparation

One of the critical structural interventions was assignment of a mentoring focal person in each regional health bureau. The focal person reports to the HIV core process owner in each region. Furthermore, the following steps were taken to enhance the transition. (1) Establishment of mentoring transition taskforce, made up of members from the regional health bureaus and I-TECH. The taskforce used terms of reference to develop CMP transition roadmap and lead the transition at the regional level. (2) Development of roadmap to define the steps to be taken to ensure the success of the transition. (3) A signed MOU between the regional health bureaus and I-TECH and (4) development of action plans setting forth specific tasks, activities, responsibilities, and timelines.

Moreover, to enhance the CMP, a strategy of joint mentoring was designed and implemented in January/February 2014. Joint mentoring was preceded by training of facility mentors on mentoring skills. Joint mentoring refers to mentorship coordinated among the regional health bureaus, facilities, and I-TECH. Its purpose was to enhance the knowledge and skill of trained government mentors (i.e., mentors from lead facilities), and instruct mentees from cluster facilities through hands-on training by I-TECH capacity building teams. It was also designed to enable government mentors to mentor and supervise the mentoring process in their own facilities. Furthermore, joint mentoring was intended to be replaced by independent mentoring by teams from lead facilities after 6 months. Independent mentoring has been in place in the three regions since July 1, 2014. Moreover, joint supportive supervision was the additional mechanism intended to strengthen the capacity of facilities through visits, discussions, and feedback.

### Regional and Facility Level Capacity

International Training and Education Center for Health conducted several training workshops intended to build local capacity. The workshops targeted regional health bureaus’ program managers, facility staff, and staff from local university. The aim is to enable trainees lead, coordinate, and manage the mentoring program, as well as mobilize and deploy resources. The training workshops covered eight specific areas: grant-writing skills, human resources development and management, project cycle management, mentoring and administrative skills, partnership coordination and management, leadership and management, asset and financial management, and USAID/CDC rules and regulations and US financial management. Most of these training activities were conducted a year before the implementation of the survey.

The facility participants appreciated the transfer of skills and knowledge to hospital staff to improve competence in clinical mentoring and build confidence of mentoring staff. Facility participants believed that clinical mentoring knowledge and skills were already acquired at facility level. Focus group discussants from the hospitals expressed it in the following way:

I say thank you I-TECH because they have shown us [how to do it]. I-TECH has done so many things including from renovation of facilitates up to assisting the technical aspects.—Focus group discussion with multidisciplinary team, hospital-A1

The regional health bureaus demonstrated their commitment by expanding the CMP beyond its original target (cluster hospitals) by establishing links between hospitals and health centers. Health centers were assigned to each hospital in the respective regions. Although this plan was not included in the collaboration between I-TECH and the regional health bureaus, it was discussed and approved by the regional health bureaus. This plan would certainly be favored by regional and national government agencies, as the expected impact would be even more significant. This is because most people do not live close to hospitals, and instead obtain health services from nearby health centers and clinics.

International Training and Education Center for Health’s capacity building teams, regional directors, and RPM&E coordinators mostly gave positive feedback regarding the scope of transition activities. This included I-TECH’s capacity building components, and the transition process itself. The majority expressed confidence that the regional health bureaus will do well in the long run, regardless of the challenges they may face.

The health bureaus are doing well. The HIV core process and mentoring focal persons have been assigned, their contact information (email addresses) has been recorded, and feedback is coming in through the reports. The bureaus are getting to know their people.—Focus group discussion with I-TECH

However, there were also concerns. Some doubted the capacity of the regional health bureaus. The capacity was not as strong as originally expected and the duration of time for the transition was insufficient.

I do not know how much I-TECH has done at regional level, but now there is one person assigned and that is not enough to run all the activities in the region. Focus group discussion with mentors, hospital-C1

It is difficult to believe the regional health people can replace them [I-TECH] because I did not see the independent mentoring. My fear is related to finance. I think the regional people may not have adequate training. Focus group discussion with multidisciplinary team, hospital-B1

### Facility Readiness to Own CMP

As evidenced from the reports and interviews, mentoring activities were conducted in all three regions. Potential mentors were recruited and trained. Findings from the focus groups with mentors and interviews with mentees showed that facilities were committed to conduct independent mentoring and were expanding the mentoring to health centers. Lead facilities have already been mentoring staff at cluster facilities since the start of the joint mentoring program.

The hospital has started owning [the program]; it is taking responsibility. The hospital and area health facilities are determined to own the clinical mentoring program, so they are helping each other to take on more responsibilities.—Focus group discussion with multidisciplinary team, hospital-A1

The mentees we interviewed were a mix of those who had been working in HIV services for quite some time, and those who joined recently. Mentees were supervised by internal mentors, by mentors from lead facilities and by I-TECH mentors as part of the joint mentoring program. It was revealed that those who were newly assigned to the HIV ward were not well informed about the transition and the quality of mentoring was not to the same standard of what I-TECH was doing.

When I-TECH handled mentoring, we would get full information, but when it [the mentoring] is [done] by facility staff, the information we get is limited, because we are receiving it second-hand [i.e., through facility mentors who get the primary information from I-TECH].—Interview with mentee, hospital-C1

The above expression suggests that newly trained facility mentors’ capacity was not on par to I-TECH mentors. This was not surprising as I-TECH staff were better equipped and had more mentoring experience.

Internal supervision was not difficult for most of the facilities with mentors. This is because internal mentors can conduct mentoring in their own facility with little disruption of their routine activities. On the other hand, when mentors travel to cluster hospitals, activities are interrupted in their facilities. Mentoring other facilities demands commitment of staff and logistical arrangements. Supervising cluster facilities were expected to be more challenging because of the additional resources required from the respective regional health bureaus. For example, it requires *per diem*, transportation, other associated expenses, and mentors’ experience. These could put facilities under administrative, logistical, and financial constraints.

Most of the hospitals are prepared for running clinical mentoring activities. Multidisciplinary team meetings were used to identify problems, provide solutions, and monitor implementation. The meetings are attended by the Chief Executive Officer (CEO), Chief Clinical Officer (CCO—equivalent to a medical director), and other hospital management personnel.

We have multidisciplinary team meetings. In these meetings, we raise and discuss all our problems. Those who provide HIV services are included in the MDT [multidisciplinary team] meetings and the medical director [CCO] and CEO of the hospital attend these meetings. So, if there is a problem, because the medical director and CEO are present, we can solve it immediately.—Interview with mentee, hospital-C1

Other hospitals have also started sending mentors to cluster facilities to provide supervision as part of joint and independent mentoring:

Our clinical mentors go down to provide support. We collect feedback, as well as observe documentation showing the activities, decisions, and actions taken. We even have these on hand here.—Focus group discussion with multidisciplinary team, hospital-A1

We triangulated the findings through reviewing documents collected from the facilities and I-TECH. We used the data abstraction format for the period covering April 2013–March 2014 with extension to August 2014. The data collection addressed mentoring, case-based discussions, multidisciplinary team meetings, and capacities.

#### Mentoring

One of the areas we searched in the documents was whether mentoring activities were conducted by I-TECH staff only, jointly with I-TECH or independent of I-TECH. In practical terms, available documents showed that mentoring activities performed before March 2014 were primarily handled by I-TECH staff. However, there were a few instances of joint mentoring indicating that most of the joint mentoring activities were started after March. We also looked at the number of remote consultations conducted between lead and cluster facilities. Communication between mentors is mainly done face-to-face but it also included written feedback, telephone calls, and email communications. According to I-TECH monthly reports, 260 distant (*via* telephone, email) consultations (an average of 18.6 per month) took place from April 2013 to May 2014 in Amhara, 102 (7.3 per month) in Afar, and 48 (3.4 per month) in Tigray. However, it was very difficult to rely on these figures because there were differences in the style of reporting in the three regions as well as within the same region.

#### Case-Based Discussions

For instance, in the Amhara region, a total of 214 case-based discussions was conducted across all facilities, but documentation for individual facilities was not available. However, based on the I-TECH regional Capacity Building Team report for COP13 (13), it appears that the case-based discussions were conducted jointly with I-TECH. In Tigray in the three hospitals included in the sample, 14 case-based discussions were conducted by facility staff. In Afar, we found no evidence of case-based discussions conducted by facility staff.

#### Multidisciplinary Team Meetings

Based on 14 months of data extracted from I-TECH’s monthly activity reports (14) (from April 2013 to May 2014), 142 MDT meetings were conducted in Amhara, 125 in Afar, and 114 in Tigray. Of these, 68% of the meetings in Tigray were conducted independently, 62% in Afar, and 41% in Amhara. Therefore, except for the Amhara region, well over 50% of multidisciplinary team meetings were conducted independent of I-TECH.

#### Number of Available Mentors

The number of trained mentors was 89 (Amhara), 35 (Afar), and 53 (Tigray). The evaluation team learned that these mentors were trained by I-TECH in response to requests from the respective regional health bureaus. One of the things that was not evaluated was the level of competence of the mentors and whether there was any transfer out of trained staff.

#### Availability of Funds

Donor funding for the CMP had already been secured for Amhara and Tigray; however, the government did not include funding for any of the three regions from its own budget. But, the Tigray and Amhara regional health bureaus have undertaken discussion on how to sustain CMP and diversify funding sources.

#### Vehicles

To address regional transportation needs, the CDC Ethiopia, Federal Ministry of Health, and the regional health bureaus have already completed a detailed analysis. The analysis encompassed regional needs, planned procurement under COP13/COP14, numbers of vehicles to be transferred from partners (I-TECH, Management Sciences for Health, Johns Hopkins University), and gaps in the system. The CDC communicated the plan to the Federal Ministry of Health through an official letter (15). According to the plan, the vehicle shortage may not be a long-term problem. However, providing sufficient number of vehicles for the facilities in the respective regions would remain a big challenge.

#### Laboratories

The WHO tools for measuring improvements in laboratory services were used to assess the status of enrollment of laboratories. These were (1) Strengthening Laboratory Measurement Toward Accreditation (SLMTA) which is a training and mentoring tool kit and (2) Stepwise Laboratory Quality Improvement Process Toward Accreditation (SLIPTA) a framework for auditing laboratory standards used for evaluation. Enrollment in the WHO–AFRO stepwise accreditation program and achievement was assessed with the attainment of one or more “stars.” The “stars” indicate their quality improvement grades based on semi-annual external SLMTA/SLIPTA assessments in a formal recognition report. Based on this, in the Afar region, three of the 21 I-TECH supported laboratories—the regional laboratory, and the laboratories at two other hospitals—earned one or more stars for quality improvement during the period from April 2013 to March 2014. In the Amhara region, 19 of the 21 laboratories were enrolled in the WHO–AFRO program. Four of them earned one or more stars for quality improvement during the same period: one earned three stars, two earned two stars, and one earned one star. We were unable to obtain aggregate data on the accreditation status of laboratories in the Tigray region. This information might have been missed during data collection because the evaluation team has not visited the regional laboratory. However, the hospitals that were visited did have documentation showing their accreditation status. Axum hospital earned two stars for quality improvement under the WHO–AFRO program; two other hospitals were enrolled in the program, but had not been graded. A fourth hospital was in the process of renovating its laboratory facilities. The renovation was near completion at the time of our visit to the hospital.

#### Point-of-Care (POC)

We assessed the presence of three or more POC for provider-initiated HIV testing and counseling sites. All 19 (2 are from non-program facilities) Amhara region hospitals had three or more POC sites. Similarly, all three facilities visited in the Tigray and Afar regions each, had three or more provider-initiated testing and counseling services. At Axum hospital, however, it was reported that most of the POC sites, except at labor and delivery units, were not functioning at the time of the study. It was mentioned that there was shortage of supplies affecting the services. Although there are limitations, it is well known that POC testing increases access to HIV diagnosis, and thus increases enrollment and participation in treatment (16).

## Discussion

In this study, we explored the CMP transition process, identifying several important points. These are documented as lessons learned. Included were critical issues that need to be expanded upon and emphasized for subsequent learning and strengthening of the program.

As discussed in the results section, the process of transferring ownership of the program was conceived by I-TECH through early discussion and transition plans with regional health bureaus and facilities’ management. The cornerstone of the transition was supported by the Global Health Initiative, PEPFAR 5-year strategy, and the Ethiopian government’s commitment to take over and integrate the CMP (5, 17–19). This indicates that the transition is within the context of the international trend of promoting country ownership and ensuring sustainability (4, 5). One of the published lessons from transitioning an HIV program to local owners is the Avahan transition strategy in India that used a logic model. The transition strategy included (1) building capacities of local community, non-governmental organizations and government sectors, (2) aligning technical capacity with government activities, and (3) measuring the outcome of the transitioning (20).

In our study, the regional health bureaus integrated the CMP into their existing structures by assigning a focal person under the HIV core process. As we learned from the regional health bureaus, it will take some time for the CMP to be incorporated into the organizational chart. The fact that the regional health bureaus were committed to integrating the CMP and its subsequent implementations illustrates their basic commitment to sustaining the program. Engagement of leaders of the regional health bureaus and facilities was facilitated by holding transition launch workshops, recruiting potential mentors, and providing training workshops. This was found to be effective in placing a focal point at the regions for enhancing the transitioning.

In collaboration with I-TECH, the regional health bureaus performed the tasks required to provide training and build capacity within a very short time—9 months (December 2013—September 2014). These activities were critical to facilitating the transition through joint and independent mentoring. The joint mentoring program gave facility mentors opportunities to learn and demonstrate their knowledge and skills to I-TECH Capacity Building Teams who provided technical assistance when necessary. The joint mentoring helped to share experience between I-TECH and governmental mentors which was important to maintain consistency and quality of the clinical mentoring. However, it was also learned that the duration of time for joint mentoring was very short and most of the activities were crammed in the last 9 months before this assessment was conducted. This might have an impact on the quality and scope of sustaining the program affecting performance and outcome. In contrast, other authors have reported that effective transition of programs to local ownership requires long term and built-in design that can be monitored and evaluated (20).

The HIV CMP requires skilled personnel. These personnel require regular follow up and updating, as well as equipment, materials, supplies, finance, good management, and coordination through documentation, and regular written feedback. Committed champions and effective leadership at the health bureaus is essential. However, it was learnt that focal persons at the regional health bureaus usually have additional responsibilities hampering fulltime and effective coordination of the CMP. As reported in other areas human resource is one of many critical challenges to sustain quality of HIV/AIDS care and treatment in rural areas of developing countries where most people live (21).

Although the transition began in December 2012, involvement on the part of the regional health bureaus and implementation of most of the transition steps (including joint mentoring) became intensive over its last 9 months (22). As indicated earlier, the delay might have its own impact in establishing an effective mentoring transition. Engaging local health authorities and other stakeholders from the beginning and subsequent follow ups is very important to make sure that ownership is firmly established. Political commitment is one of the nine core domains to indicate ownership and sustainability of public health programs as reported by some authors (23). Furthermore, effective transitions are inclusive involving government, communities, non-governmental organizations, and the private sector (20). However, in our study, involvement of other providers such as private hospitals and non-governmental organizations was not observed.

Most of I-TECH’s activity took place at the health facilities. Creating a steadily improving learning environment and building mentoring capacity at facility level was a good practice. After all, most of the activities of the mentoring program take place at health facilities and respective managements. If the necessary support is provided to facilities by the regional health bureaus, our findings suggest the CMP could be sustainable. Furthermore, data and information are generated from multidisciplinary team meetings and case-based discussions, which are conducted regularly (previously, meetings were held sporadically). In addition, mentoring teams at the facility level have well-developed experience in implementing the CMP.

Facilities are also service delivery points. They have a better grasp of day-to-day services and have immediate implications on patients. The regional health bureaus’ idea of extending the provision of clinical mentoring to health centers was widely accepted, as the impact would be visible and significant in the regions. This would also be attractive to the regional governments, as it would have wide, grassroots coverage. However, it would also be challenging, as this expanded model will require significant financial, logistical, human resource, and administrative support from the regional health bureaus and lead hospitals. It may be relevant to mention that the expanded program would be compromised if it lacks adequate emphasis and proper support. It is also important that the federal ministry of health provides its support as the experiences would benefit other regions and similar programs.

One of the strengths of the hospitals was their ability to effectively participate in the joint mentoring activities. This ultimately led to their progressing to independent mentoring at most facilities before I-TECH involvement was phased out. This was confirmed in our review of reports and documented feedback. The progress made by the hospitals has contributed significantly to improving the capabilities of government mentors. However, there was also a planning-time constraint as there was not enough time to observe and learn from the independent mentoring.

Although the study found considerable improvements in data management, much remains to be done to make it available and useful for improving services at the facility level.

The reports of case-based discussions and multidisciplinary team meetings were good examples of record keeping, and using the data to improve services. However, there were gaps and inconsistencies in this regard with serious deficiencies noted in Afar region. It may be relevant to note that Afar is an “emerging region” with poor infrastructure and with critical shortage of health work force even by Ethiopian standards. In Tigray, the data collection team might have missed searching for the report during their visit to the regional health bureau. If the problems in documentation and reporting are not addressed, analysis of data at regional health bureau and ministry of health level would remain difficult affecting its utilization. Use of simplified formats harmonizes and improves feedback.

Except for the status of accreditation carried out by the Ministry of Health using the WHO-AFRO protocol, the capacity of laboratory services was not assessed in detail. As observed from the results, most of the laboratories are not at satisfactory level of accreditation. As building and maintaining laboratory quality is resource intensive, changing the *status quo* will be a major challenge for the respective regional health bureaus and facilities.

Shortage of vehicles was one of the frequently cited challenges to expanding and sustaining an effective CMP. With the expansion of the program to include health centers and establishing a strong inter-facility mentorship, obtaining logistical support is fundamental. As demonstrated from the discussions and interviews most mentors regarded this as one of the urgent problems to be dealt with. One good sign was that CDC Ethiopia (donor) and the Federal Ministry of Health had concrete plans for alleviating the vehicle shortage. However, under the plan, about a quarter of the demand for vehicles will not be met. In the long run, therefore, the burden may fall on hospitals, as many health centers fall under the oversight of each hospital leading to increased pressure on resources and logistical services.

Financial problems in general would have considerable impact on the CMP. Hospital services could be compromised if sufficient funds are not allocated and disbursed in a timely manner. This would also reduce the commitment of management and staff. Finances are also necessary to prevent shortages of materials and supplies, both of which are essential to improving the program. Additionally, keeping knowledge and skills up to date requires financial support to the facilities by the respective regional health bureaus. Since funding has already been secured for a few years, the focus would be on how to distribute the money to the facilities where mentoring is being implemented. Furthermore, efforts to incorporate CMP in the budget of hospitals in the region should start step-by-step so that the program would be able to continue after the support is withdrawn.

One critical challenge that may be beyond the reach of facilities is shortage of skilled personnel/mentors. Shortages may happen because of staff turnover, inadequate staffing of health facilities, and growing demand for health services in local communities, which will increase the workload for facilities and staff. This problem can be solved by the regional health bureaus, as it is possible to assign new staff to facilities facing shortages, and respond quickly to such acute problems. However, other competing priorities of the regional health bureaus do exist. This will partially affect the degree of emphasis on the HIV CMP affecting implementation of the entire program. This was appreciated during our discussions with the focal persons of the regional health bureaus and staff and management of facilities. One of the strategies used by I-TECH as part of local capacity building in the transition was involvement of regional universities. It was reported that University of Gondar (Amhara region) and Mekelle University in Tigray have progressed well in this regard. Responding to training needs could be evident using the existing collaboration between the regional health bureaus and universities (24).

In general, ownership of the CMP by facilities was viewed positively by participants, with most appreciating its value and showing commitment to sustaining it. This was evident in the engagement of most staff and management in multidisciplinary team meetings, mentoring, case-based discussions, and integration of the program by the regional health bureaus and facilities into their existing organizational structures.

This study has the following limitations: first, the transition period was short and therefore did not evaluate program outcomes. Second, the study did not include authorities from the Federal Ministry of Health and Federal HIV/AIDS Prevention and Control Office. These offices could have provided ideas regarding the directions of transitioning, envisaged constraints, and strategies to enhance sustainability of the program at country level. Third, the fact that the study is limited to a few hospitals in each region may not reflect the status of transitioning at remaining facilities located in remote areas. However, we had also the opportunity to attend a review workshop in a region where many of the representatives participated. We accessed reports that helped us appreciate commonalities and specific problems in the facilities and regional health bureaus. Fourth, there was limited literature regarding transitioning of clinical mentoring.

## Conclusion and Recommendations

The capacity building process for transitioning the CMP was largely effective, more so at the facility level. This could lessen the level of effort needed from the regional health bureaus to increase awareness and build capacity to accommodate the program.

The regional health bureaus have made significant progress in taking ownership of the CMP in the three regions. However, much remains to be done in terms of structure, coordination, and responding to the needs of facilities, to ensure the gains made are sustained.

The expansion of the CMP to health centers was a huge undertaking by the regional health bureaus. While the merit of this expansion is obvious, it is resource intensive. Already serious gaps are evident that require urgent attention.

As the transition period was short, quality of care outcomes related to the CMP in transition could not be evaluated. However, as quality of care indicators are incorporated in the CMP, outcomes can be evaluated at the appropriate time. Along the same line, the WHO–AFRO stepwise accreditation process for the laboratories should continue and be monitored to track improvements. There are critical challenges to CMP that will have negative effects if not addressed in a timely manner. These include problems related to transportation, *per diem*, materials and supplies, equipment maintenance, and staff/mentor shortages.

We recommend (1) strengthening of the structure (mentoring focal unit) of the regional health bureaus through recruiting additional experts including people with experience in mentorship to provide leadership and oversight, (2) ensuring continuity of the multidisciplinary teams, case-based discussions, continuous quality improvement activities, and catchment area meetings, (3) ensuring the availability of qualified mentor pool, (4) as needed, involve non-governmental organizations and provide strong administrative support at regional and facility levels, (5) strengthening of program monitoring, written feedback using simplified and uniform formats for improved documentation, and (6) designing of a stepwise approach to obtain government resources allocation.

## Author Contributions

GK contributed in the design, tool development, data collection, analysis of data, and write up of the manuscript. TB contributed in the design, tool development, analysis of data, and critical review of the manuscript. AS contributed in the design, tool development, analysis of data, and write up of the manuscript. GF contributed in the design, critical review, and write up of the manuscript.

## Conflict of Interest Statement

The authors declare that the research was conducted in the absence of any commercial or financial relationships that could be construed as a potential conflict of interest.
